# Capsaicin as a Microbiome Modulator: Metabolic Interactions and Implications for Host Health

**DOI:** 10.3390/metabo15060372

**Published:** 2025-06-05

**Authors:** Iván Artemio Corral-Guerrero, Angela Elena Martínez-Medina, Litzy Yazmin Alvarado-Mata, Ana Cristina Figueroa Chávez, Roberto Muñoz-García, Miriam Paulina Luévanos-Escareño, Jazel Doménica Sosa-Martínez, María José Castro-Alonso, Padma Nimmakayala, Umesh K. Reddy, Nagamani Balagurusamy

**Affiliations:** 1 Laboratorio de Biorremediación, Facultad de Ciencias Biológicas, Universidad Autónoma de Coahuila, Torreón 27000, Mexico; ivan.corral@uadec.edu.mx (I.A.C.-G.); angela.medina@uadec.edu.mx (A.E.M.-M.); litzy.alvarado@uadec.edu.mx (L.Y.A.-M.); ana.figueroa@uadec.edu.mx (A.C.F.C.); roberto_munoz@uadec.edu.mx (R.M.-G.); miriam_luevanos@uadec.edu.mx (M.P.L.-E.); jsosa@uadec.edu.mx (J.D.S.-M.); castro_maria@uadec.edu.mx (M.J.C.-A.); 2 Department of Biology, Gus R. Douglass Institute, West Virginia State University, Institute, Dunbar, WV 25112-1000, USA; padma@wvstateu.edu

**Keywords:** capsaicin, microbiota, host–microbiome interactions, nutritional modulation

## Abstract

**Background/Objectives:** Capsaicin is the principal pungent compound in chili peppers and is increasingly recognized as a multifunctional phytochemical with systemic effects beyond its sensory properties. It has been linked to metabolic regulation, neuroprotection, inflammation control, and cancer modulation. This review aims to provide an integrative synthesis of capsaicin’s metabolism, its interaction with the gut microbiome, and its physiological implications across organ systems. **Methods:** We conducted a critical literature review of recent in vivo and in vitro studies exploring capsaicin’s metabolic fate, biotransformation by host enzymes and gut microbes, tissue distribution, and molecular pathways. The literature was analyzed thematically to cover gastrointestinal absorption, hepatic metabolism, microbiota interactions, and systemic cellular responses. **Results:** Capsaicin undergoes extensive hepatic metabolism, producing hydroxylated and dehydrogenated metabolites that differ in transient receptor potential vanilloid type 1 (TRPV1) receptor affinity and tissue-specific bioactivity. It crosses the blood–brain barrier, alters neurotransmitter levels, and accumulates in brain regions involved in cognition. In addition to its systemic effects, capsaicin appears to undergo microbial transformation and influences gut microbial composition, favoring short-chain fatty acid producers and suppressing pro-inflammatory taxa. These changes contribute to anti-obesity, anti-inflammatory, and potentially anticancer effects. Dose-dependent adverse outcomes, such as epithelial damage or tumor promotion, have also been observed. **Conclusions:** Capsaicin represents a diet-derived bioactive molecule whose systemic impact is shaped by dynamic interactions between host metabolism and the gut microbiota. Clarifying its biotransformation pathways and context-specific effects is essential for its safe and effective use in metabolic and neurological health strategies.

## 1. Introduction

Capsaicin is the primary pungent alkaloid found in chili peppers (*Capsicum* spp.), widely consumed as both a culinary ingredient and a traditional remedy [1]. Its physiological relevance has transcended its sensory role, as numerous studies have demonstrated capsaicin’s capacity to modulate metabolic, inflammatory, neurological, and oncogenic pathways [2]. These properties have established capsaicin as a promising dietary phytochemical with broad pharmacological potential. However, its biological activity is highly context-dependent and influenced by multiple variables, including dose, tissue distribution, route of administration, and, critically, its metabolic fate within the host [3]. Upon ingestion, capsaicin is rapidly absorbed in the gastrointestinal tract and subjected to extensive first-pass hepatic metabolism, mainly by cytochrome P450 enzymes such as CYP2C9, CYP2C19, and CYP3A4 [3,4]. These reactions result in hydroxylated and dehydrogenated metabolites that vary in their affinity for the TRPV1 receptor and their biological effects. Recent studies have shown that capsaicin is also able to cross the blood–brain barrier, accumulate in lipid-rich brain regions (striatum, cerebellum, and brain stem), and undergo in situ metabolic transformations, affecting neurotransmitter dynamics and neuromodulatory functions [4].

In parallel, emerging evidence highlights the gut microbiota as a crucial determinant of capsaicin’s metabolic fate and systemic impact [3]. Capsaicin, in turn, modulates microbial community structure, often favoring the growth of beneficial taxa such as *Ruminococcaceae*, *Lachnospiraceae*, and *Faecalibacterium*, while reducing pro-inflammatory *Proteobacteria* [5,6]. Moreover, capsaicin has been shown to regulate gene expression linked to apoptosis (*STAT3*), oxidative stress (*RUNX2*), thermogenesis (*UCP1*), and epigenetic mechanisms [7,8,9,10]. These molecular effects support its application in models of obesity, inflammation, neurodegeneration, and cancer. However, depending on concentration and duration of exposure, capsaicin may also elicit adverse effects such as mucosal damage or pro-tumorigenic responses in specific contexts [11].

This review aims to provide a comprehensive and integrative synthesis of capsaicin’s metabolic pathways both host and microbiota-mediated its systemic bioactivity, and the factors that influence its pharmacological potential. Special attention is given to its transformation in the liver and brain, its modulation by the intestinal microbiota, and its downstream effects on metabolic, immunological, and neurological functions. By exploring these interconnected processes, this article seeks to clarify the role of capsaicin as a dietary bioactive compound within the broader context of host–microbiome–metabolite interactions.

## 2. Biosynthesis, Consumption, and High-Pungency Pepper Varieties

### 2.1. Capsaicin Biosynthesis

Pungency in peppers is caused by capsaicinoids, simple alkaloids present in the *Capsicum* species. The two most abundant capsaicinoids are capsaicin and dihydrocapsaicin, accounting for over 90% of the present capsaicinoids. Capsaicinoids are important for plants, insects and animals. Due to the presence of a phenolic group in its structure, it can act as an antioxidant, protecting the plant from biotic and abiotic stress [12]. Capsaicinoids also present positive effects in the human body, serving as antioxidants and providing anti-inflammatory, antimicrobial, anticancer, anti-virulence, analgesic, and antidiabetic properties [13].

**Figure 1:**
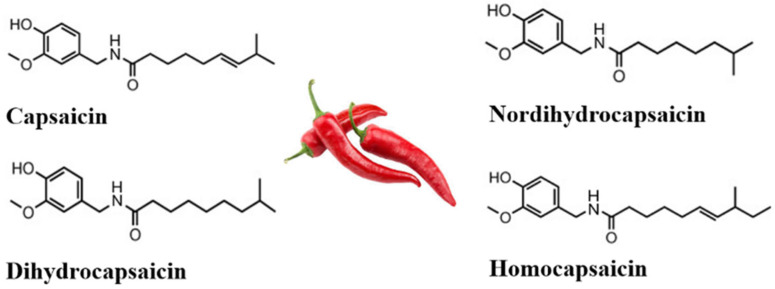
Chemical structures of the main capsaicinoids present in *Capsicum* spp. fruits: capsaicin, dihydrocapsaicin, nordihydrocapsaicin, and homocapsaicin. These molecules possess a vanilloid nucleus linked to an aliphatic chain by an amide bond. Named (E)-N-(4-hydroxy-3-methoxybenzyl)-8-methyl-6-nonenamide (capsaicin), N-(4-hydroxy-3-methoxybenzyl)-8-methylnonanamide (dihydrocapsaicin), N-(4-hydroxy-3-methoxybenzyl)-7-methyloctanamide (nordihydrocapsaicin), and (E)-N-(4-hydroxy-3-methoxybenzyl)-9-methyl-6-decenamide (homocapsaicin) according to the IUPAC nomenclature. Variations in the length and degree of unsaturation of the aliphatic chain affect their hydrophobicity and pungent potency.

Capsaicin (C_18_H_27_NO_3_) is a natural alkaloid present in *Capsicum* plants (Figure 1) [14]. Its molecular structure consists of a benzene ring connected by a polar amide group to a long hydrophobic carbon chain [7], making it highly lipophilic. Chili peppers synthesize capsaicin as a defense mechanism against herbivores and microbial threats, causing a ‘burning’ sensation in animals and insects, deterring them from approaching or consuming food from that area, and altering the ovipositional behavior of insects [15]. Its distribution within the fruit is uneven, with approximately 89% concentrated in the placenta, while only 5–6% is present in the pericarp [1].

Capsaicin is a potent agonist perceived by TRPV1, a transmembrane cation channel that functions with Ca^2+^. It possesses six putative transmembrane domains and a calcium-permeable pore region. TRPV1 possesses a vanilloid-binding pocket located in the transmembrane domain of the channel, where capsaicin binds and allosterically alters its properties, which causes an increase in Ca^2+^ flux, changing the electrical properties of the cell and provoking the release of neurotransmitters that are involved in the signaling of pain and inflammation, such as substance P and calcitonin gene-related peptide (CGRP) [16]. Moreover, humans are not the only TRPV1 containers; other vertebrates such as rodents, frogs, rabbits, and chickens have been studied for a better understanding of these receptors [17]. Other organisms like the fruit fly *Drosophila melanogaster* are used as transgenic models [18].

**Figure 2:**
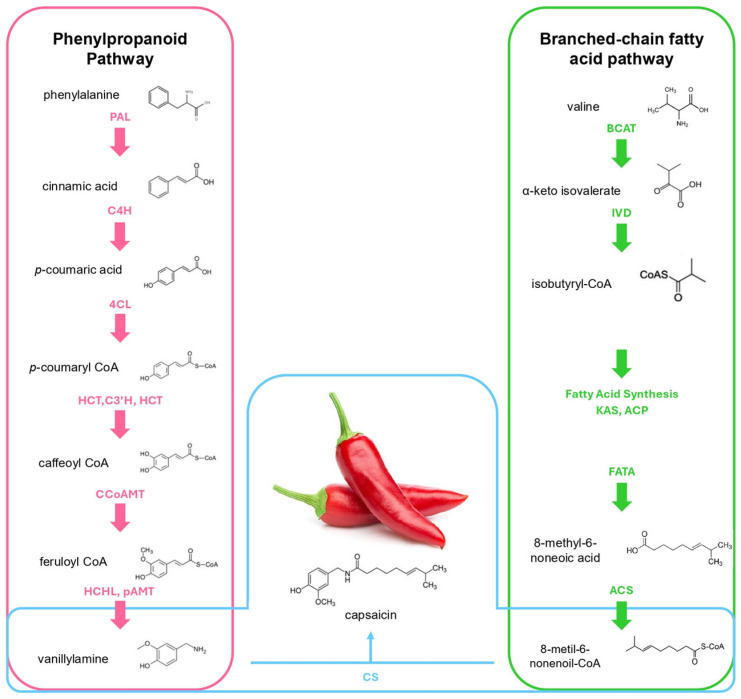
Metabolic routes involved in capsaicin synthesis. The phenylpropanoid pathway and the branched-chain fatty acid pathway are linked. The phenylpropanoid pathway provides the aromatic moiety, while the branched-chain fatty acid pathway contributes the fatty acid component. These two precursors are ultimately joined to form capsaicin. The enzymes participating in the phenylpropanoid pathway in order: phenylalanine ammonia-lyase (PAL), cinnamate-4-hydroxylase (C4H), 4-hydroxycinnamate CoA ligase (4CL), hydroxycinnamoyl CoA: shikimate hydroxycinnamoyl transferase (HCT), coumaric acid 3-hydroxylase (C3′H), caffeic acid O-methyltransferase (CCoAOMT), 4-hydroxycinnamoyl-CoA hydratase/lyase (HCHL), putative amino transferase (pAMT). The enzymes participating in the BCFAP in order: branched-chain amino acid aminotransferase (BCAT), isovaleryl dehydrogenase (IVD), β-ketoacyl-ACP synthase (KAS), acyl carrier protein (ACP), fatty acyl-ACP thioesterase A (FATA), capsaicin synthase (CS).

The biosynthesis of capsaicin (Figure 2) primarily occurs through two metabolic pathways, mainly in the placenta of epidermal cells of pepper fruit; the phenylpropanoid metabolic pathway (PMP) and the branched-chain fatty acid pathway (BCFAP). A key precursor in this process is 4-Coumaroyl-CoA, which is synthesized via the PMP. The pathway begins with the deamination of phenylalanine to cinnamate by phenylalanine ammonia-lyase (PAL), which is further converted by the action of cinnamate 4-hydroxylase (C4H), yielding *p*-coumarate, transformed into *p*-coumaroyl CoA catalyzed by 4-hydroxycinnamate CoA ligase (4CL). *p*-coumaryl shikimate is obtained by the action of hydroxycinnamoyl CoA: shikimate hydroxycinnamoyl transferase (HCT), and the action of coumaric acid 3-hydroxylase (C3′H) produces caffeoyl shikimate, which is transformed by a caffeic acid O-methyltransferase (CCoAOMT) to produce feruloyl CoA, which is further converted to vanillin by 4-hydroxycinnamoyl-CoA hydratase/lyase (HCHL). Finally, a putative amino transferase (pAMT) converts it to vanillylamine [7].

Fatty acid chains are synthesized within plastids in both leaves and developing seeds through the action of a multi-enzyme fatty acid synthase (FAS) complex [19]. This complex catalyzes the elongation of fatty acyl intermediates, which are covalently linked to acyl carrier protein (ACP). The precursor, acetyl-CoA, is primarily derived from the breakdown of photosynthates via glycolysis in the cytosol. Subsequently, pyruvate dehydrogenase complexes located in both mitochondria and plastids convert pyruvate into acetyl-CoA. Acetyl-CoA is first converted into malonyl-CoA by the action of acetyl-CoA carboxylase (ACC). Malonyl-CoA is then transferred to acyl carrier protein (ACP) by malonyl-CoA:ACP transacylase, forming malonyl-ACP. Subsequently, β-ketoacyl-ACP synthase III (KAS III) catalyzes the condensation of malonyl-ACP with acetyl-CoA to produce 4:0-ACP. Fatty acid chain elongation continues with KAS I, which extends 4:0-ACP to 16:0-ACP (palmitoyl-ACP), followed by KAS II, which further elongates it to 18:0-ACP (stearoyl-ACP) [20]. The saturated 18:0-ACP is then desaturated by stearoyl-ACP desaturase (SAD), introducing a double bond to form 18:1-ACP (oleoyl-ACP). Finally, fatty acyl-ACP thioesterase A (FATA) and fatty acyl-ACP thioesterase B (FATB) hydrolyze the acyl-ACP intermediates, releasing free fatty acids. These fatty acids exit the plastid and are converted into acyl-CoA, forming a cytoplasmic acyl-CoA pool that serves as a precursor for lipid biosynthesis [21].

The synthesis of branched-chain fatty acids begins with the precursor valine, which is converted into α-ketoisovalerate by the action of branched-chain amino acid aminotransferase (BCAT). This intermediate is then processed by isovaleryl dehydrogenase (IVD), yielding isobutyryl-CoA. Subsequently, fatty acid synthesis, mediated by KAS and ACP, leads to the formation of 8-methyl-6-nonenoic acid through the action of FATA. Further modification by desaturase transforms this compound into 8-metil-6-nonenoil-CoA. Finally, acyl-CoA synthetase (ACS) catalyzes the conversion of 8-methylpentanoic acid into 8-methyl-6-nonenoyl-CoA, a key precursor in the biosynthetic pathway [22]. Lastly, capsaicin is synthesized by a capsaicin synthase (CS).

### 2.2. Chili Pepper Consumption

**Figure 3:**
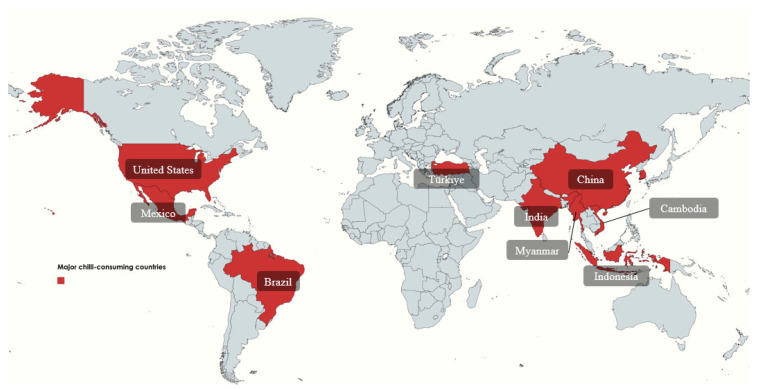
Global distribution of major chili consumers. The map highlights countries with high chili consumption, including Mexico, India, China, Thailand, Turkey, and the United States, among others. These regions reflect culinary traditions that incorporate chili peppers as essential ingredients, influenced by cultural preferences, climate, and local agricultural practices.

Chili pepper is a globally recognized spice that has been an integral part of gastronomy for centuries, consumed both as a vegetable and a condiment. Native to Central and South America [23], chili peppers have been a staple in indigenous diets since at least 7000 BC and played a significant role in religious and cultural ceremonies. The domestication of wild chili plants began approximately 5000 years ago, leading to significant morphological modifications. Later, Christopher Columbus introduced chili peppers to Europe, where they rapidly spread and became a key ingredient in various cuisines [24]. While Mexico remains one of the principal consumers of chili peppers, countries such as China, South Korea, Japan, and Thailand are also among the world’s top producers and consumers (Figure 3).

Mexico is a global leader in green chili production, ranking between second and fourth place worldwide, with a record-breaking 3.2 million tons produced in 2023, up from 3.08 million tons in 2021 and 3.1 million tons in 2022. Cultivation expanded to 165,226 ha in 2023, with Sinaloa, Chihuahua, and Zacatecas contributing 59.7% of national production. Other key producers include San Luis Potosí (324,870 tons), Sonora (187,591 tons), Guanajuato (145,362 tons), Jalisco (140,253 tons), and Baja California Sur (83,121 tons). Green chili represents 19.4% of Mexico’s vegetable production, with a per capita consumption of 15.7 kg and an annual growth rate of 3.3% (2013–2022), supporting exports to 47 international markets. Mexico supplies 8% of the world’s chili consumption, with the United States as the largest importer, generating over USD 1.04 billion in 2022, followed by Canada, Spain, the UK, Germany, the Netherlands, Japan, Israel, Costa Rica, Guatemala, and China [25].

### 2.3. Pepper Varieties

The *Capsicum* genus comprises around 35 species; this genus is one of the most diverse within the *Solanaceae* family, with 38 recognized species. Among them, the most widely cultivated worldwide are *C. annuum*, *C. frutescens* (cayenne or red pepper), *C. pubescens* (rocoto and manzano peppers), *C. chinense* (habanero-type peppers), *C. baccatum* (ají amarillo), and *C. assamicum*. Of these, *C. annuum* is the most economically significant, encompassing bell peppers, paprikas, pimentos, and the majority of Mexican chili varieties [26]. Additionally, several exotic species are produced in smaller quantities, including *C. buforum*, *C. campylopodium*, *C. cardenasii*, *C. ceratocalyx*, *C. chacoense*, *C. coccineum*, *C. cornutum*, *C. dimorphum*, *C. dusenii*, *C. eximium*, *C. flexuosum*, *C. friburgense*, *C. galapagoense*, *C. geminifolium*, *C. havanense*, *C. hookerianum*, and *C. hunzikerianum* [27]. Pungency is experienced as a heating or biting sensation and is measured using the Scoville Heat Scale, which quantifies the capsaicin content in chili peppers to determine their spiciness [28]. The scale ranges from 0 to 16 million Scoville Heat Units (SHU), with pure capsaicin at the highest rating. Based on this scale, peppers are classified into five categories: non-pungent (0–700 SHU), mildly pungent (700–3000 SHU), moderately pungent (3000–25,000 SHU), highly pungent (25,000–70,000 SHU), and very highly pungent (above 80,000 SHU) [1].

Chili peppers have long held an important place in diverse cultures around the world, not only serving as a main ingredient in culinary traditions but also carrying deep religious and symbolic meanings. Beyond their cultural and gastronomic significance, the unique chemical composition of chili peppers presents a wide range of opportunities for medical applications. As research continues to reveal new therapeutic potentials, chili peppers are increasingly recognized not just as a source of flavor and tradition but also as a promising reservoir of natural compounds for innovative treatments.

## 3. The Impact of Gut Microbiome in Host Health

Phytochemicals are bioactive compounds that we consume in our diet through fruits and vegetables. A correlation has been reported between the consumption of phytochemicals and the modulation of the gut microbiome, demonstrating an impact on human health [29]. Capsaicin is a bioactive compound of chili peppers responsible for their spicy flavor, which also shows antioxidant, anti-obesity, analgesic, anti-inflammatory, anticarcinogenic, and cardioprotective effects [30]. This phytochemical has been used as a therapeutic agent in chronic painful conditions (e.g., diabetic and non-diabetic neuropathy, osteoarthritis, or rheumatoid arthritis) [31,32,33,34,35]. However, in recent years, a topic of study is the effect of the interaction of capsaicin and the gut microbiome on human health [36]. 

**Figure 4:**
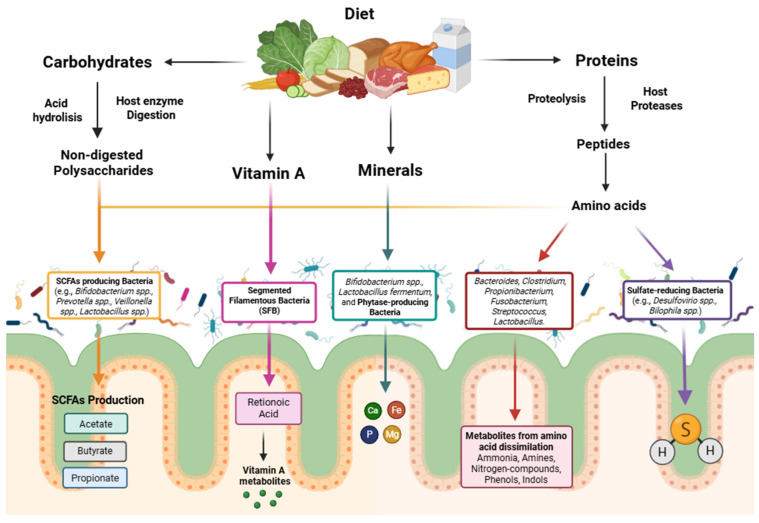
Dietary macro-, and micronutrients metabolism by gut microbiome. Complex polysaccharides that were not digested in the small intestine such as inulin, resistant starch, or galacto-oligosaccharides are fermented by gut bacteria (*Bifidobacterium*, *Prevotella* spp., *Veillonella* spp., *Lactobacillus* spp.) through specific metabolic pathways (acetogenesis, succinate, acrylate, propanediol pathways), generating short-chain fatty acids (SCFAs) which strengthen the intestinal barrier, regulate the immune system, and modulate inflammation. Amino acids are metabolized in the colon by bacteria such as *Bacteroides*, *Clostridium*, *Desulfovirio* spp., and *Bilophila* spp.; the main processes are transamination, deamination, and decarboxylation of amino acids producing SCFAs, ammonia, nitric oxide, polyamines, and phenolic and indole acids, which in low concentrations are beneficial for intestinal homeostasis. Vitamins and minerals are essential for the maintenance of host health, although gut microorganisms (e.g., *Bifidobacterium*, *Bacteroides*, *Enterococcus*) synthesize de novo essential vitamins (e.g., K, riboflavin, niacin, B12); Segmented Filamentous Bacteria transform dietary vitamin A into active retinoids, which are essential for maintaining immune homeostasis and epithelial function; and phytase-producing bacteria release minerals from food by producing phytases, which hydrolyze the phytic acid present in vegetables, releasing minerals such as calcium, magnesium, iron, and phosphorus, improving their bioavailability to the host. On the other hand, the production of SCFAs reduces the pH of the intestine, improving the solubility of minerals and facilitating their absorption through the intestinal epithelium. Created with Biorender.com.

The human microbiome is a complex microbial community residing in several body locations and can be divided into gut, oral, respiratory and skin microbiomes, playing a crucial role in improving human well-being [37]. The gut microbiome contains about 100 trillion microorganisms primarily composed of bacteria, but it is also possible to find yeast, archaea, and viruses; each person has a unique microbiome profile. This community of microorganisms is formed in the early stages of life, between 4 and 36 months of a person’s life, and the richness and diversity formed in this period will determine the optimal composition of the healthy gut microbiome for each individual [38]. Nevertheless, the gut microbiome is considered the principal one in terms of the impact on human health; important biological functions for the wellness of the host such as modulation of nutrient metabolism or regulation of the immune system are responsible for the mutually beneficial relationship between the microorganisms inhabiting the gastrointestinal tract and the host [39], since the intestine provides nutrients to the resident microorganisms, while they contribute to the digestion and absorption of these nutrients (e.g., non-digested polysaccharides, proteins, vitamins, and minerals) (Figure 4) [40].

Factors such as diet and geographic location can directly impact gut microbiome composition [41]. Differences in the composition, diversity, and enterotypes of the human gut microbiome between countries have been reported. In Asia, the predominant enterotype is *Bifidobacterium*, while in the Americas and Europe it is *Bacteroides*, and in African populations it is *Prevotella* [42]. However, it has not been determined whether the impact on microbiome composition is due to genetics or cultural and behavioral factors (lifestyle, dietary habits, environmental exposure, among others) [43]. 

The alteration in the composition and function of the intestinal microbiome has an impact on the development of diseases; therefore, a disturbed microbiome or dysbiosis is linked with the host’s disease [39]. In addition to the fact that diet has an impact on the composition of the intestinal microbiome [44], this community of microorganisms may be the key to explaining the physiological effects of diet on health and therefore on the development of chronic diseases [45]. In individuals who carry out a plant-based and Mediterranean diet, the production of short-chain fatty acids increases, as well as trimethylamine N-oxide (TMAO) levels decrease, helping to prevent cardiovascular diseases; however, in individuals with a Western diet, there is a negative alteration of the microbiome and the opposite occurs—TMAO levels increase and the production of short-chain fatty acids decreases, favoring the development of metabolic diseases [44]. This indicates that dietary interventions based on the profile of the intestinal microbiome can be implemented for the treatment of metabolic, cardiovascular, and other diseases, including foods rich in fiber and phytochemicals (e.g., capsaicin, polyphenols, curcumin), which positively influence the health of the host [46].

It could be thought that the maturation of the gut microbiome is a random process; however, this is not accurate. The gestational age of the individual, the mode of birth, and breastfeeding are factors that determine the development of the microbiome, which from an early age is participating in the metabolism of vitamins, iron, and amino acids—molecules necessary for metabolic and immune health, brain development and impact on the behavior of the individual [47]. Therefore, the first 1000 days of the host’s life are the origin of the development of health and disease [48].

Gut dysbiosis early in life has been associated with an increased risk of developing allergic diseases and obesity. Low gut microbial diversity contributes to the development of immune-mediated diseases, such as asthma or allergic rhinitis, which may persist into adulthood [48,49]. A healthy microbiome plays a key role in the maturation of the immune system; it modulates the activity of helper T cells, promotes the formation of regulatory T cells, and participates in the production of immunoreactive metabolites, such as butyrate. These mechanisms help prevent sensitization to allergens and reduce the likelihood of developing allergies and asthma [50,51,52]. In particular, children born by cesarean section or who received multiple antibiotic treatments during infancy show a higher prevalence of asthma, as well as an elevated abundance of bacteria of the phylum *Proteobacteria* [53]. 

Two dominant phyla account for 90% of the composition of the gut microbiome: *Firmicutess* and *Bacteroidetes*, and only 10% is composed of bacteria from other phyla (*Actinobacteria*, *Proteobacteria* and *Verrucomicrobia*). Although the profile of the gut microbiome will be unique for each individual, the ratio between the two main phyla (*Firmicutes/Bacteroidetes*) is used as a biomarker of an imbalance in the gut microbiome and is related to obesity, since it is associated with the fermentation of dietary polysaccharides to short-chain fatty acids (SCFA), which play a role in gut barrier function and appetite regulation and provide 10% of the total dietary energy supply in humans [54].

Obesity results when energy intake exceeds energy expenditure, and because the microbiome plays a role in energy homeostasis through multiple interdependent mechanisms such as sharing bioenergetic resources (aromatic amino acids, B vitamins, SCFAs, heme, menaquinones), extracellular electron transport, homeostasis of vitamins and cofactors, and elimination of toxins and harmful metabolites [55,56]. Studies have been carried out in different populations, where it is reported that obese people present a higher level of *Firmicutes* and a lower level of *Bacteroidetes* [54,55]; this imbalance is associated with diets high in carbohydrates and fats, which promote intestinal dysbiosis. Also, bacteria of the phylum *Firmicutes* are efficient in fermenting fiber and polysaccharides, generating SCFAs such as acetate, which can promote lipogenesis [57]. However, factors such as diet and physical activity are relevant in the diagnosis of obesity, so future studies should improve the characterization of the population to allow personalized interventions and treat obesity through direct manipulation of the intestinal microbiome [58]. 

Type 2 diabetes mellitus (DM2) is a disease characterized by elevated blood glucose levels, and it is projected that by 2045, 1 in 8 adults will be living with this disease. In recent years, advances in high-throughput sequencing and bioinformatics have provided us with solid information on the relationship between the gut microbiome profile and metabolic diseases, such as DM2 [59].

Through metagenomic analysis, species-level microbial strains and their functions associated with DM2 have been identified; certain strains of *Escherichia coli* showed virulence factors and horizontal gene transfer mechanisms, suggesting a microbial evolution that could be related to type 2 diabetes. In addition, strains of *Prevotella copri* and *Eubacterium rectale* show an increased ability to synthesize branched-chain amino acids, metabolites linked to insulin resistance and diabetes, and also possess genes associated with motility and chemotaxis, which may confer adaptation to an intestinal environment with oxidative stress and inflammation [60]. The development of further studies that analyze the microbial profile at the strain level to understand the mechanisms associated with DM2 will allow the development of targeted therapies and biomarkers. 

Intermediate metabolites or end products synthesized by the gut microbial community, just like short-chain fatty acids, tryptophan catabolites, or secondary bile acids [61], act as receptor ligands in human cells, having a direct impact on health, protecting against inflammatory bowel diseases and neurological conditions [62].

Crohn’s disease and ulcerative colitis are inflammatory bowel diseases characterized by symptoms of diarrhea, abdominal pain, and weight loss. In recent years, the active participation of the intestinal microbiome in metabolic health is related; the microbial profile between healthy individuals and individuals suffering from inflammatory bowel diseases differs, presenting a lower proportion of *Firmicutes* such as *Lactobacillus*, *Roseburia*, *Ruminococcus*, *Lachnospir* and *F. prausnitzii*, which produces a protein that suppresses ulcerative colitis and butyric acid, essential for intestinal epithelial cells [63]. As well as an increased presence of sulfate-reducing gamma and *Deltaproteobacteria*, *Enterococcus*, *Megasphaera*, *Campylobacter*, and *Fusobacterium*, revealing metabolic pathways related to inflammation and linked to sulfur metabolism, bacterial toxin secretion systems, and purine metabolism. For example, sulfate-reducing bacteria by producing hydrogen sulfide exhibit acyl-CoA dehydrogenase, an enzyme related to butyrate oxidation, which causes dysbiosis in the intestine [64].

Similarly, lipids produced by the host microbiome, such as SCFAs, sphingolipids, phospholipids, sulfolipids, among others, act as stimulators of mucosal and systemic immunity. They primarily interact in the small and large intestine, influencing immunity through pattern recognition receptors (PRRs) (e.g., TLR4, TLR2, NLRs, CLRs), immune cells (e.g., NKT cells, macrophages, dendritic cells), and cytokines (e.g., IL-12, IL-23, IL-1β). The impact they have varies depending on the lipid structure [65]. Individuals with lower gut microbiome diversity are linked to a higher level of short-chain fatty acids and are therefore more prone to cardiometabolic diseases such as glycemia, dyslipidemia, and hypertension, due to the presence of microbial strains that are associated with these diseases: *Enterobacter hormaechei*, *Haemophilus parainfluenzae*, *Streptococcus*, and *SMB53*, in contrast to those individuals who show a lower level of short-chain fatty acids, show a diverse microbiome and with beneficial strains [66].

The microbiome not only has an impact on the gastrointestinal tract and diseases related to this part of the body; it also plays a role in the central nervous system, having a bidirectional communication microbiota–gut–brain, indicating the impact of the community of microorganisms in neurological processes through the vagus nerve and the modulation of the immune system, the hypothalamic-pituitary-adrenal (HHA) axis, and the metabolism of tryptophan [67]. One of the most common mental disorders globally is depression; despite the fact that 280 million people suffer from this disorder, its pathogenesis is uncertain. Due to the association of the intestinal flora with central nervous system processes, the microbial profile has been investigated in individuals with depression, the association between thirteen microbial taxa with symptoms of depression, since they are involved in the synthesis of key neurotransmitters in depression, such as glutamate, GABA and butyrate. *Sellimonas*, *Eggerthella*, *Lachnoclostridium* and *Hungatella* were genera reported in greater abundance, and *Coprococcus*, *Subdoligranulum* and *Ruminococcaceae* were found less frequently [68].

The most common cause of dementia is Alzheimer’s disease, which is a disease that causes a gradual deterioration of cognition. Dysbiosis in the intestinal microbiome also has an impact on the pathological processes in individuals with Alzheimer’s disease [69]. Comparing the microbial profile of Alzheimer’s patients, there is a decrease in microbial diversity and changes in the taxonomic composition in different bacterial genera compared to healthy patients. The two dominant phyla show a variation; *Firmicutes* is found in lower presence and *Bacteroidetes* in higher colonization, the latter producing lipopolysaccharides that can trigger systemic inflammation and are linked to amyloid pathology in Alzheimer’s disease [70]. A decrease in *Bifidobacterium*, an *Actinomycetota* with anti-inflammatory properties that reduces intestinal permeability, is also reported. Some bacterial genera belonging to the gut microbiome are related to cerebrospinal fluid (CSF) biomarkers that indicate the presence or development of neurological diseases. A high value of the biomarker p-tau/Aβ42 is closely related to the diagnosis of Alzheimer’s disease, and a higher abundance of *Bacteroides* and *Blautia* presents higher levels of p-tau/Aβ42 [71], and lower levels of Aβ42/Aβ40 in the CSF, indicating higher amyloid accumulation [72].

Another brain-affecting disease is Parkinson’s disease (PD), a neurodegenerative disorder impacting both the central and peripheral nervous systems, leading to cognitive and motor decline [73]. This condition is strongly linked to gut microbiome alterations, where patients show disrupted bacterial diversity characterized by increased abundance of species like *Bifidobacterium dentium*, *Actinomyces oris*, and *Streptococcus mutans*, alongside decreased beneficial bacteria such as *Roseburia intestinalis* (a producer of anti-inflammatory short-chain fatty acids). The microbiome also shows elevated levels of opportunistic pathogens (*Escherichia coli*, *Klebsiella pneumoniae*) that promote a proinflammatory environment through immunogenic molecules (e.g., LPS) and toxic metabolites like trimethylamine. These changes coincide with dysregulation of 30–67% of metabolic pathways, reducing production of key neurotransmitters (dopamine, glutamate, GABA, serotonin) while increasing degradation of intestinal proteins and mucins, collectively exacerbating systemic inflammation and disease progression [74]. 

Finally, neurodevelopmental disorders such as autism spectrum disorder affect brain development, resulting in individuals with poor communication skills, limited reasoning, and repetitive behavior patterns. Studies suggest that genetics and environmental factors are involved in the pathology of autism spectrum disorder (ASD); however, in recent years, the intestinal microbial community has been associated with ASD [75,76,77]. In individuals with ASD, there is a decrease in *Bifidobacterium* related to alterations in the metabolism of tryptophan, a precursor of serotonin. This bacteria at low levels is associated with metabolic alterations and gastrointestinal symptoms. Similarly, there is an increase in *Clostridia*, *Sarcina* and *Parabacteroides* linked to production of neuroactive metabolites and toxins that accentuate the symptomatology of ASD [78].

An altered gut microbiome influences the development of endocrine, immunological, and neurological pathologies. Dysbiosis in the intestine causes changes in bacterial metabolism contributing to systemic inflammation and signal transduction pathways through the production of metabolites related to the development of certain host pathologies [37]. Therefore, strategies such as the consumption of probiotics and prebiotics, fecal matter transplant from healthy donors, as well as a balanced diet, exercise and adequate rest will allow the improvement of the gastrointestinal microbiome, therefore a better health for the host [70].

## 4. The Role of Host Metabolism and the Gut Microbiome in Capsaicin Biotransformation

Capsaicin is primarily absorbed in the gastrointestinal tract via passive diffusion, with the stomach and jejunum identified as the principal sites of uptake. Absorption rates in animal models are notably high, ranging from 85% to 95% following oral or intragastric administration. However, capsaicin exhibits low systemic bioavailability due to its rapid metabolism in the liver and other tissues, resulting in a short plasma half-life of approximately 25 min in humans [3].

Following absorption, capsaicin undergoes extensive phase I hepatic metabolism, predominantly catalyzed by cytochrome P450 enzymes, including CYP2C9 and CYP2C19. These enzymes facilitate hydroxylation of the aliphatic side chain, producing 16-hydroxycapsaicin and 17-hydroxycapsaicin as primary metabolites [3,4]. A third metabolic route involves alkyl dehydrogenation, yielding 16,17-dehydrocapsaicin, a metabolite with increased irritancy due to its retained high-affinity binding to TRPV1, the primary receptor mediating the pungent and neuroactive effects of capsaicin [4].

Interestingly, molecular docking simulations have demonstrated that while 16,17-dehydrocapsaicin retains strong binding to TRPV1 via hydrogen bonds at residues L515 and T550 similar to native capsaicin, 16-hydroxycapsaicin and 17-hydroxycapsaicin exhibit altered orientations that reduce their ability to activate TRPV1 [4,79]. This suggests the possibility that the metabolic conversion of capsaicin may serve as a detoxification mechanism that diminishes its pungency and biological stimulation in peripheral tissues.

In terms of tissue distribution, capsaicin shows preferential accumulation in the kidneys, liver, heart, and lungs, with relatively lower concentrations in the brain and spleen after systemic administration [4]. This rapid distribution highlights the hydrophobic character of capsaicin, which facilitates membrane diffusion and extensive tissue binding, but also accelerates its clearance.

Importantly, recent in vivo studies on Sprague-Dawley rats employing blood–brain synchronous microdialysis have confirmed that capsaicin and its metabolites rapidly cross the blood–brain barrier due to its lipophilic nature via passive diffusion, with 17-hydroxycapsaicin being the predominant brain metabolite. Notably, the profile of capsaicin metabolism in the brain differs from that in the liver, suggesting region-specific enzymatic activity and possible in situ metabolism [3,4]. These findings are particularly relevant given the emerging interest in capsaicin’s neuroactive effects, including modulation of dopamine, acetylcholine, GABA, and serotonin.

Overall, capsaicin’s metabolic fate is shaped by both host enzymatic activity and its physicochemical properties, which together constrain its systemic bioavailability. Understanding these processes is essential not only for evaluating its pharmacological potential but also for developing formulations or delivery systems such as encapsulation or co-administration with bioenhancers capable of optimizing its therapeutic index.

### 4.1. Role of Gut Microbiota in Capsaicin Metabolism

While hepatic metabolism is well established as the primary pathway for capsaicin biotransformation, the gut microbiota is increasingly recognized as a complementary metabolic system that modifies the chemical structure, bioactivity, and systemic availability of dietary capsaicin. The microbiota, particularly in the anaerobic environment of the colon, possesses a wide array of enzymatic activities that can alter xenobiotics, including plant-derived compounds [80]. Evidence from analogous phytochemicals and polyphenols suggests that gut bacteria can transform capsaicin through reductive cleavage, dehydroxylation, demethylation, and conjugation reactions, although direct identification of capsaicin-specific microbial metabolites remains limited. These microbial transformations likely occur prior to hepatic processing, or even in parallel, particularly in individuals with slower gastrointestinal transit or high microbial enzymatic activity [7,81].

Capsaicin’s long aliphatic chain and vanillyl group provide potential substrates for microbial enzymatic attack. It is hypothesized that aromatic ring-cleaving enzymes, similar to those seen in polyphenol metabolism, may generate vanillyl-based derivatives, while hydroxylases and reductases may act on the amide bond or side chain, contributing to the formation of bioactive or less pungent metabolites. These modifications may decrease capsaicin’s affinity for TRPV1 or change its absorption kinetics. Although most current in vivo evidence comes from hepatic and systemic studies, indirect indicators support the role of the microbiota. For example, individuals or animal models with different microbiota compositions display marked differences in plasma metabolite profiles after capsaicin ingestion. This is further supported by observations that capsaicin metabolites such as 16- and 17-hydroxycapsaicin may be partially produced or pre-processed in the gut before hepatic circulation [4,82].

**Figure 5:**
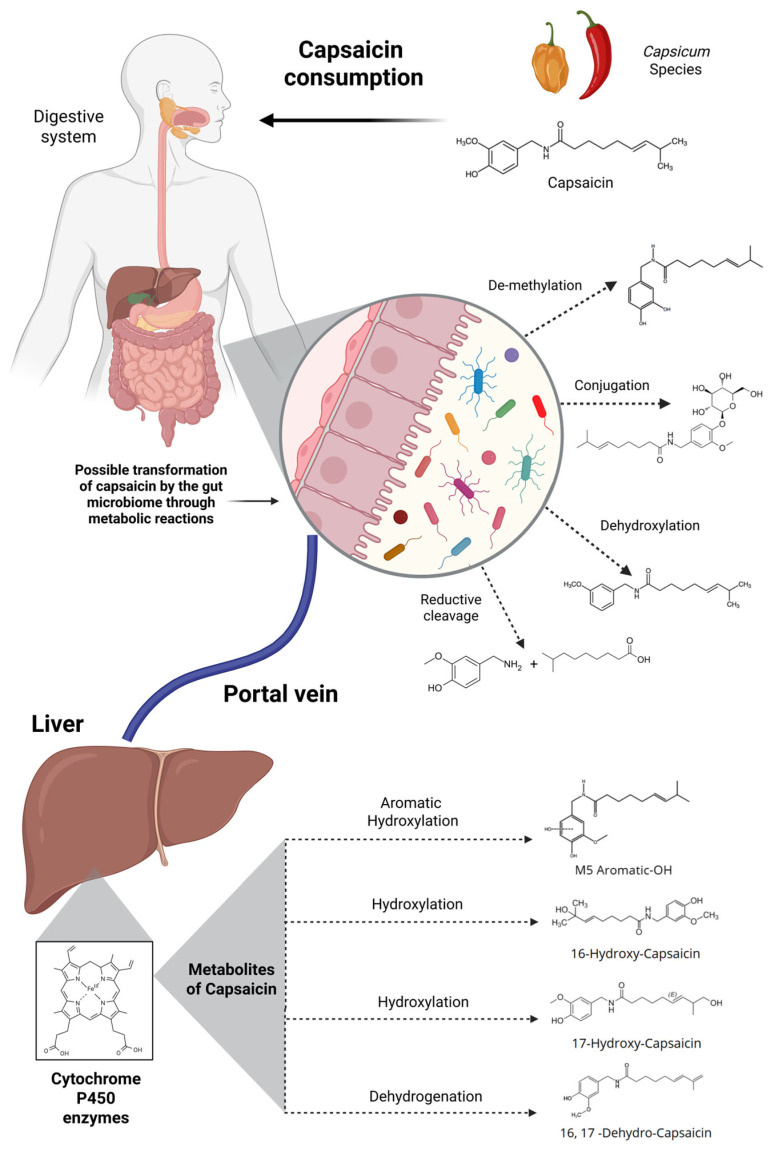
Proposed microbial contribution to capsaicin biotransformation. After ingestion, capsaicin may undergo enzymatic modification by gut microbiota, including demethylation, conjugation, dehydroxylation, and reductive cleavage, potentially altering its systemic bioavailability or affinity for TRPV1. These microbial processes may act prior to hepatic metabolism via the portal vein. Hepatic cytochrome P450 enzymes further convert capsaicin into hydroxylated and dehydrogenated metabolites. Created with Biorender.com.

Moreover, in animal models, capsaicin metabolism may exhibit enterohepatic recycling (only <10%), where the glucuronidated form is secreted in bile and is re-exposed to gut bacteria (e.g., β-glucuronidases) [83,84]. This microbial recycling could prolong systemic exposure or alter local colonic effects. Advanced techniques such as stable isotope tracing and mass spectrometry imaging offer promising tools to resolve the relative contributions of microbial vs. hepatic pathways. However, these approaches have not yet definitively mapped microbial metabolites of capsaicin, highlighting a critical gap in knowledge. To illustrate the proposed microbial contributions to capsaicin metabolism, Figure 5 provides a conceptual overview.

### 4.2. Factors Influencing Capsaicin Bioavailability

Capsaicin’s bioavailability is determined by multiple interrelated factors, including its physicochemical properties, metabolic transformations, route of administration, and the biological context of the host, including gut microbiota composition. Despite high absorption efficiency in the gastrointestinal tract reported to exceed 85% in animal studies, capsaicin exhibits low systemic bioavailability primarily due to its lipophilic nature and extensive first-pass hepatic metabolism. Once absorbed, capsaicin is rapidly metabolized in the liver by cytochrome P450 enzymes, especially CYP2C9, CYP2C19, and CYP3A4, producing hydroxylated and dehydrogenated metabolites such as 16-hydroxycapsaicin, 17-hydroxycapsaicin, and 16,17-dehydrocapsaicin. These biotransformations not only limit the amount of active compound available in circulation but also alter its pharmacological profile, as certain metabolites show reduced interaction with the TRPV1 receptor [3,85].

The route and formulation of administration significantly affect capsaicin’s absorption and systemic delivery. Oral ingestion typically leads to substantial first-pass metabolism, while transdermal or topical application can bypass hepatic processing, though absorption in these cases is constrained by the skin’s barrier function. To overcome solubility and stability limitations, various advanced formulations have been developed. For instance, polymeric micelles, liposomes, and hydroxypropyl-β-cyclodextrin complexes have demonstrated the capacity to enhance capsaicin’s oral bioavailability, prolong its plasma half-life, and improve therapeutic consistency [3,86].

Host factors also play a substantial role. Variability in hepatic enzyme activity driven by genetic polymorphisms, sex, age, and health status can influence the rate and extent of capsaicin metabolism, leading to interindividual differences in drug exposure and response. Additionally, gut microbiota contributes to capsaicin biotransformation through microbial enzymatic activity in the intestinal lumen and by modulating enterohepatic recycling. After hepatic conjugation, capsaicin metabolites can be secreted into the bile and subjected to deconjugation by microbial β-glucuronidases, enabling their reabsorption and prolonging systemic availability [3,85,87]. Moreover, microbial communities may directly modify capsaicin via reductive or hydrolytic pathways, although these transformations remain poorly characterized and represent an important area for future investigation.

Lastly, the tissue distribution of capsaicin and its metabolites is influenced by its hydrophobicity and affinity for lipid-rich tissues. In vivo studies have shown high accumulation in the kidneys, liver, and heart, with lower but significant levels detected in the brain. The presence of capsaicin and its derivatives in brain regions associated with reward and memory suggests that tissue-specific pharmacokinetics could contribute to its neuromodulatory effects. Overall, capsaicin bioavailability is constrained by chemical, physiological, and microbial factors but may be optimized through the design of effective delivery systems and the consideration of host-specific variables.

### 4.3. Capsaicin Metabolism and the Central Nervous System

Recent research has revealed that capsaicin, once absorbed into the bloodstream, is not only distributed systemically but also capable of crossing the blood–brain barrier and undergoing distinct metabolic transformations within the central nervous system. In contrast to peripheral metabolism, which is dominated by hepatic CYP450-mediated hydroxylation and dehydrogenation reactions, capsaicin in the brain exhibits a unique pharmacokinetic profile characterized by delayed elimination and regional accumulation of specific metabolites [4,88]. Following peripheral administration, capsaicin was detected in multiple brain regions, including the striatum, hippocampus, cortex, cerebellum, and brainstem, with peak concentrations observed within 30 to 50 min. Notably, higher levels were measured in lipid-rich regions such as the brainstem and cerebellum, suggesting that lipophilicity plays a key role in its neural distribution.

Among the metabolites identified, 17-hydroxycapsaicin was found to be the dominant product in brain tissue, in contrast to 16-hydroxycapsaicin, which is more prevalent in the liver. This indicates that capsaicin undergoes in situ hydroxylation within the brain, likely catalyzed by brain-expressed CYP enzymes such as CYP2C9 and CYP2D6. These findings suggest that capsaicin metabolism in the central nervous system is not merely a reflection of hepatic processing but involves local enzymatic activity that may shape region-specific effects [4].

Metabolites formed in the brain exhibit different affinities for the TRPV1 receptor. While 17-hydroxycapsaicin shows reduced interaction with TRPV1, the dehydrogenated metabolite 16,17-dehydrocapsaicin retains strong binding affinity and may contribute to capsaicin’s characteristic irritant and excitatory effects on sensory neurons. Molecular docking studies have demonstrated the binding capability of this metabolite to key activation sites such as Thr550, Tyr511, and Leu515 on TRPV1 [89], similar to native capsaicin, whereas hydroxylated metabolites adopt alternative orientations that avoid these binding residues [4,90].

Functionally, capsaicin and its metabolites significantly influence neurotransmitter dynamics in the brain. Following acute administration, capsaicin increases dopamine levels in reward-related areas such as the striatum and cortex and enhances acetylcholine release in the hippocampus and cortex regions associated with memory and cognitive function. In contrast, it inhibits acetylcholine release in the cerebellum and olfactory bulb. These region-specific responses suggest that capsaicin may modulate brain activity through both TRPV1-dependent and independent pathways [91]. Additionally, capsaicin exposure alters glutamate, GABA, and serotonin levels in distinct brain regions, with potential implications for neuroprotection, mood regulation, and energy metabolism.

Chronic or repeated capsaicin administration has also been linked to broader changes in brain metabolic pathways. Untargeted metabolomics analysis revealed that repeated capsaicin exposure upregulates amino acid metabolism and downregulates purine catabolism in the brain. These changes may contribute to reduced oxidative stress and neuroinflammation, supporting proposed benefits of capsaicin in neurodegenerative disease models, including Alzheimer’s and Parkinson’s disease [91,92].

Taken together, these findings highlight the capacity of capsaicin to reach the central nervous system, undergo local metabolism, and exert region-specific effects on neurotransmission and metabolic homeostasis. Future studies should further explore how capsaicin’s neuropharmacological properties may be leveraged for therapeutic purposes, and whether its effects can be modulated through microbiota-mediated interactions, genetic variability in metabolic enzymes, or formulation strategies that enhance brain targeting.

## 5. Effects of Capsaicin Intake on the Diversity and Composition of Gut Microbiota

**Figure 6:**
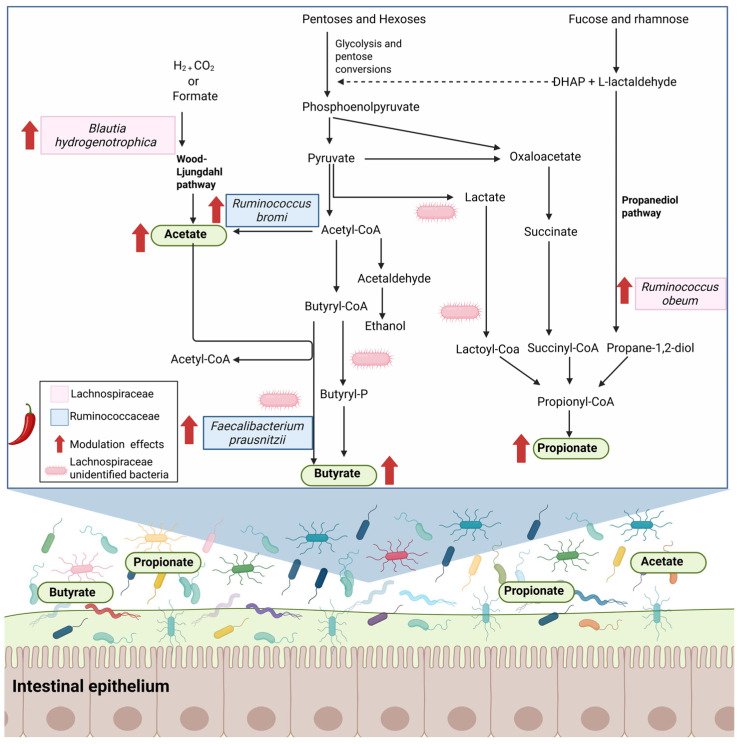
Proposed microbial pathways for SCFA biosynthesis modulated by dietary capsaicin in the gut microbiome. The diagram illustrates key pathways by which gut microbiota metabolize carbohydrates to generate the major short-chain fatty acids (acetate, butyrate, and propionate). Pink ovals represent members of the *Lachnospiraceae*, including unidentified bacteria. Blue boxes represent taxa from *Ruminococcaceae* and pink boxes from *Lachnospiraceae*. Red arrows indicate increased abundance from capsaicin modulation. Created with Biorender.com.

It is well known that components in diet are one of the external factors that modulate the composition and function of gut microbiota [93]. The gut microbiota is one of the densest microbial communities in the body, and several studies have demonstrated its role in host health [94]. In this context, recent studies have investigated the interaction between capsaicin and intestinal microbiota. Two main mechanisms have been most postulated: (1) In mice, capsaicin has been associated with an increase in the diversity of bacteria that produce short-chain fatty acids (SCFA) such as butyrate, propionate, and acetate (Figure 6) that are associated with epigenetic regulation and anti-inflammatory signaling [95]. Butyrate and propionate produced by microbial fermentation, have been reported to inhibit the activity of histone deacetylase enzyme in both intestinal epithelial cells and immune cells; this epigenetic inhibition negatively regulates the expression of pro-inflammatory cytokines in immune cells including colonic macrophages [96,97,98]. (2) There is also evidence that capsaicin reduces the abundance of certain gram-negative bacteria producing lipopolysaccharide (LPS), which are major components of the bacterial outer membrane [99]. These LPS can interact and activate the TLR4 receptor (Toll-like receptor 4), whose activation triggers a signaling cascade that promotes the transcription of proinflammatory cytokines (e.g., interleukin-1β, IL-8 and tumor necrosis factor-α) through the transcription factor NF-κB resulting in an inflammatory response in the intestinal epithelium and damage to the intestinal barrier [100].

For example, in a wildtype male mouse, a capsaicin-enriched diet can prevent microbial dysbiosis induced by high-fat diets; this effect is associated with an increase in the abundance of short-chain fatty acid-producing bacteria, such as *Ruminococcaceae* and *Lachnospiraceae*, as well as a reduction in both the abundance of lipopolysaccharide-producing bacteria and the expression of genes involved in their biosynthesis [6].

Similarly, capsaicin demonstrated intestinal anti-inflammatory effects by decreasing the abundance of lipopolysaccharide-producing bacteria and lipopolysaccharide-induced inflammatory cytokines related to *Proteobacteria* phylum such as Helicobacter, Desulfovibrio and Sutterella, in TRPV1-knockout (TRPV1−/−) mice [101].

The administration of capsaicin in the diet was also related to an increase in the *Faecalibacterium* genus in the intestinal microbiota; however, this effect was shown to be sex-dependent. In male mice, it gradually increased with gastric perfusion, while in female mice, this effect disappeared [95]. A study in humans analyzed the effects of capsaicin based on gut enterotypes. Two enterotypes were identified; Enterotype 1 (E1), characterized by a predominance of *Bacteroides*, and Enterotype 2 (E2), dominated by *Prevotella*. The study found that the effects of capsaicin on the microbiota were more pronounced in individuals with the E1 enterotype. Compared to E2, E1 individuals showed a higher abundance of the genus *Faecalibacterium*, a genus associated with intestinal butyrate production, and was linked with higher butyrate concentration in feces [5]. These results were independent of sex, in contrast to the previous study in rats.

Another study in male C57BL/6J mice reported that capsaicin increased levels of short-chain fatty acids depending on the dose; butyric acid increased at doses of 60 and 80 mg/kg, and propionic acid at 80 mg/kg, while acetic acid decreased at 60 mg/kg. A dose of 80 mg/kg increased the abundance of *Actinobacteria*, *Proteobacteria*, *Bifidobacterium*, *Faecalibaculum*, and *Butyricimonas*, which are related to the production of butyric acid or neuropeptides. However, at this concentration, it also caused intestinal damage and inflammation [30].

Previous studies showed that treatment with capsaicin (0.01%) for 9 months decreased body weight and was associated with an increase in the proportion of *Akkermansia muciniphila* in the gut microbiota [102]. This bacterium is found in the mucosal layer of the gut and is associated with increased intestinal mucus thickness and improved intestinal barrier function by reducing inflammation [103]. Interestingly, this mechanism was found to be TRPV1-dependent, as TRPV1 activation is proposed to stimulate the expression of mucins MUC2 and MUC3 in the colon, thereby increasing mucus secretion and providing carbon and nitrogen sources for the growth of *A. muciniphila* [104]. However, the complete mechanism is not fully understood.

In contrast, a recent study found that 12 weeks of a capsaicin-rich diet (300 mg/kg) disrupted intestinal barrier integrity. Unlike the previous study, a decrease in *Akkermansia* was observed, alongside an increase in mucolytic bacteria capable of weakening the intestinal barrier and causing inflammation such as *Muribaculaceae* [105].

In other mammalian animals, such as calves, capsaicin increases the abundance of *Collinsella*, a bacterium associated with the production of secondary bile acid ursodeoxycholic acid (UDCA) and with protective effects against intestinal inflammation [106]. Specifically, UDCA can inhibit the growth of pathogenic bacteria, and clinical studies have also indicated that UDCA may benefit patients with intestinal dysbiosis by modulating the intestinal microbiota [107].

In *Drosophila melanogaster* chili diets, especially those with high capsaicin such as Serrano, significantly modified microbial diversity. The proportion of the phylum *Firmicutes* (*Firmicutes*) increased in flies fed diets containing chili. Interestingly, it was shown that the changes were a combined effect and the flavonoids and carotenoids in chili modulated microbiota and not only capsaicin [108].

Previous studies have shown that the effects on changes in the observed microbiota and their impact on host health are not only due to the presence and concentration of capsaicin in the diet. Other important factors include the method of administration, diet composition, duration of exposure, organism, sex and the initial enterotype of the host microbiota. Therefore, it is important to analyze which bacterial groups can be used as markers to determine host health, since bacteria associated with certain benefits can also have negative effects depending on unknown variables [109].

On the other hand, several studies have examined the effect of capsaicin delivery on communication between the gut microbiota and the brain (microbe–gut–brain axis) [110]. Evidence suggests that altering this communication may contribute to neurodegenerative disorders [111].

For example, oral administration of capsaicin in APP/PS1 mice (Alzheimer’s disease models) increased the phylum *Verrucomicrobiota*, particularly *Akkermansia muciniphila*. This change in the gut microbiota was positively correlated with an increase in tryptophan-related metabolites, such as Kyn, a precursor of kynurenic acid, which is associated with neuroprotection by reducing oxidative stress. A reduction in lipid metabolism was also observed, particularly in metabolites such as lysophosphatidylcholines, which are associated with inflammation. These results together were associated with improved cognitive function in mice [112].

It has been shown that capsaicin present in gochujang increased gut microbial diversity in model rats with parasympathetic nervous system dysfunction; it increased the abundance of *Akkermansia* and *Blautia* genus and showed a decrease in *Clostridium* and *Romboutsia*. These changes in the intestinal microbiome improved insulin sensitivity and reduced inflammation, suggesting a protective effect mediated by capsaicin through metabolic functions such as insulin secretion, cAMP signaling and others [113].

Regulation of the composition and diversity of microbiota with capsaicin may be a strategy to treat various health problems such as obesity and cognitive diseases. Nevertheless, discrepancies observed across studies, such as sex-dependent responses in animal models that are not consistently replicated in humans, highlight the complexity of capsaicin–microbiota interactions. These variations could be explained by differences in baseline microbiota composition, genetic background, hormonal environments, and specific experimental conditions including the route of administration and diet composition.

The mechanisms by which capsaicin modulates the gut microbiota remain poorly understood and represent a significant gap in the field. While many studies have reported modulation in microbial composition following capsaicin administration, few have focused on providing mechanistic insights [114]. Existing proposals are often speculative or based on indirect evidence, with limited use of integrative methodologies that could clarify cause–effect relationships. Future studies should further explore the underlying molecular mechanisms of capsaicin-induced microbial changes. Metabolomic and transcriptomic analyses could provide deeper insight into how capsaicin modulates microbiota composition, metabolic outputs, and their interaction with host physiology.

Additionally, important knowledge gaps remain, including the long-term stability of microbiota changes induced by capsaicin and their sustained effects on host health. Addressing these issues through longitudinal studies is essential for developing targeted dietary interventions that can leverage the therapeutic potential of capsaicin effectively.

## 6. Capsaicin’s Impact on Gene and Protein Modulation

Capsaicin has attracted attention due to several activities that could benefit health, such as anti-obesity, anti-inflammatory, anti-oxidative, and anticancer activities. These properties have demonstrated capsaicin’s role in gene regulation, allowing the inhibition of adipogenesis, increasing the expression of glycolytic enzymes such as phosphoglycerate mutase and triosephosphate isomerase that has been seen getting upregulated in intestinal epithelial cells, and it also has been observed that the enhancement of energy metabolism by dietary capsaicin, which gives this role as an anti-obesity compound [115,116]. Moreover, it has been seen that capsaicin modulates cell growth, which can be related to its anticancer and anti-tumoral activity that can help to inhibit the proliferation of gastrointestinal cancer cells and promote their apoptosis; for example, hMOF that induces cell growth inhibition in gastric cells by the acetylation of the histone H4K16; however, this and other mechanisms will be discussed in detail in the following sections [117,118]. Some of these activities are related to the activation of the transient receptor potential vanilloid-1 (TRPV1) channel with which capsaicin interacts; however, it is still under discussion. TRPV1 is a channel with six transmembrane domains and a calcium-permeable region, which when interacts with capsaicin it leads to a calcium influx and depolarization; this phenomenon has an important role in cancer because of its cell-signaling related to cell death, due to the altered expression levels of TRPV1 in cancer cells. On the other hand, TRPV1 has been proven not to be related to the anti-obesity effects but only capsaicin; however, this receptor has shown that it can regulate energy expense and cholesterol metabolism [16,115,119]. In this section, it discusses the role and effects that capsaicin has on several diseases and how its consumption impacts the overall health of the individual.

### 6.1. Capsaicin’s Role in Cancer

Several studies have tested and discussed the potential utilization of capsaicin as therapeutic alternatives against cancer proliferation with the induction of cell death that has been previously reported on STAT3, MAPK, hedgehog, β-catenin, among other signaling pathways and regulation of certain genes and proteins that have a crucial role within this issue that will be discussed in this section [120]. Some of the cancer diseases that have been addressed using cell line cultures or model organisms such as mice are stomach cancer, colorectal cancer, bladder cancer, breast cancer, lung cancer, prostate cancer, among others [121]. There are different mechanisms of cell death. Typically, apoptosis is the main mechanism by which capsaicin induces cell death in cancer cells. This process begins when capsaicin triggers a calcium flux within the cell via TRPV1, activating the p38 pathway. This leads to an imbalance in intracellular calcium, causing mitochondrial calcium overload. As a result, reactive oxygen species (ROS) are produced, along with depolarization of the mitochondrial membrane potential and opening of the mitochondrial permeability transition pore. Consequently, cytochrome c is released, the apoptosome is assembled, and caspases are activated, ultimately leading to cell death [122].

**Figure 7:**
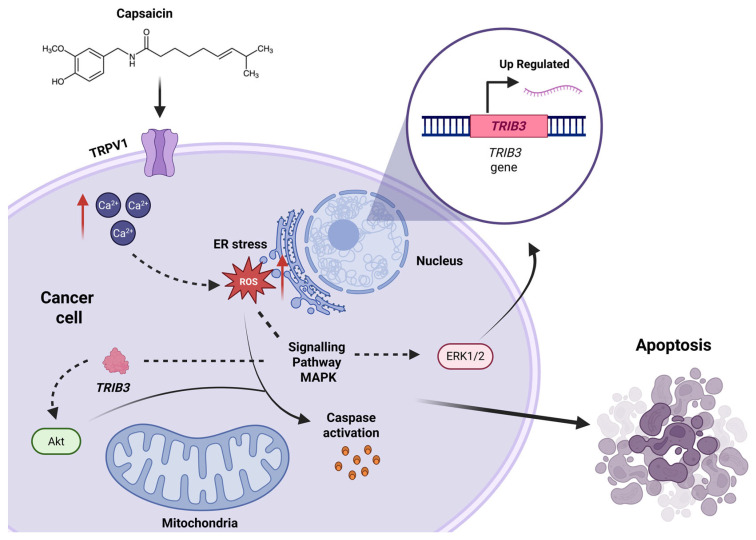
TRIB3 is a multifunctional protein scaffold-like regulator of various signaling pathways acting as a molecular switch to regulate the activation of three classes of MAPK signaling cascades; capsaicin upregulates its expression, which increases cell death rate from cancer cells. Created with Biorender.com.

However, several signaling pathways and proteins that are involved in crucial steps are often addressed as being modulated, upregulating their expression or inhibiting their activity. Tribbles-related protein 3 (TRIB3) has a role in the antitumoral activity of capsaicin in humans and its function as apoptosis regulation [123]; during this study, they tested a set of different cancer cell lines and achieved to identify that capsaicin enhances *TRIB3* gene expression, which allowed an increase in the antiproliferative and proapoptotic effects of TRIB3 in cancer cells and contributed to the understanding of TRIB3’s role in cancer cell death as shown in Figure 7.

Capsaicin can act as an inhibitor of lysine-specific demethylase 1A (KDM1A/LSD1) [124], an enzyme that functions as a histone demethylase capable of H3K4me1/2 and H3K9me1/2. KDM1A/LSD1 is known to be overexpressed in a wide variety of cancers and is associated with promoting cancer cell progression. Capsaicin was shown to inhibit KDM1A/LSD1 with an IC_50_ of 0.6 ± 0.0421 μM in a gastric cancer cell line, indicating that capsaicin can effectively inhibit cancer cell proliferation. As mentioned above, capsaicin has been seen as a phytochemical that could target certain enzymes that take charge of histone methylation or acetylation, which could take place to address diseases such as cancer that come from epigenetic issues during the regulation of gene expression in cell proliferation that end up related to tumor development or to cause other diseases; thus, it is important to address this area and to characterize the role of the enzymes that when in the presence of capsaicin, their expression or enzymatic activity gets regulated. For example, the characterization of the role of hMOF, a histone acetyltransferase for H4K16, took place during the induction of cell growth inhibition in a gastric cancer cell line when it was reactivated with capsaicin interaction, aiming to suggest that the anticancer activity of capsaicin can be related as well with the activation of hMOF [118].

In the study of expression of histone deacetylases and toll-like receptors (TLR) [125], the role of TLR is to activate innate and adaptive immunity, and its irregular activation may lead to disturbance in immune homeostasis of intestines, among other problems that result in an important regulator in colorectal cancer; thus, in this study, they looked at the effect of capsaicin and cold exposure in order to analyze its role during colorectal cancer progression, and they observed that the long-term administration of capsaicin and the cold exposure impact negatively, thereby aggravating the colorectal cancer of the rat. This negative impact of capsaicin will be later discussed, because capsaicin has been previously addressed as a “double-edged sword” in the literature.

Capsaicin has also been seen to downregulate and inhibit tumor-associated NADH oxidase (tNOX) and Sirtuin1 (SIRT1) in multiple cancer cell lines such as bladder cancer, which led to reduced cell growth and migration [126].

In hepatocellular carcinoma, capsaicin has been found to promote apoptosis and autophagy, a different mechanism of cell death; capsaicin has been identified to promote pathways that trigger apoptosis such as caspase-3 activation, signal transducer and activator of transcription-3 (STAT3) upregulation, or tNOX inhibition. For autophagy, it has been found that it induces it by p62 accumulation, activating AMP-activated protein kinase, stimulating light chain 3B (LC3-II) conversion, which places capsaicin as a phytochemical that decreases cancer proliferation and angiogenesis, a key process during cancer and tumor development [8].

A potential use of capsaicin as an anticancer nutraceutical has been tested targeting DNA topoisomerases, where their role is to regulate conformational changes that occur during DNA replication, transcription or repair, pointing out that capsaicin had an inhibitory effect on topoisomerases I and II, causing a reduction in metabolic activity and proliferation of a human colon cancer cell line, which established it as a potential approach for the antitumor effect that capsaicin has [127].

An important process during most of the colorectal cancer cells is the activity of the β-catenin/T-cell factor pathway, which is active at a high level, which was observed that with capsaicin, the β-catenin transcription gets downregulated and decreases its protein stability; this led to identifying β-catenin/T-cell factor transcription as a target of capsaicin, which proved its antitumoral activity [128].

### 6.2. Capsaicin’s Role in Liver Disease

The liver takes an important role in the human body, accounting for several activities such as energy metabolism, detoxification, drug metabolism, among others; for this reason, liver diseases catch enormous attention, and the search for novel therapies or therapeutic agents is crucial in this issue, and capsaicin has enough research that can handle it, such as drug-induced liver injury, alcoholic and non-alcoholic liver injury, liver fibrosis, among others that are related to them or cancer as discussed earlier [85].

Liver fibrosis is a disease that can into worse scenarios later on. Hepatic Stellate Cells (HSCs) play a major role in this problem, having a function in repair and matrix remodeling and being a major contributor to the development of this disease. Capsaicin in this case results as a key agent for modulating HSC activation; along with that, it also decreases the activation features of already active HSCs and is able to inhibit the initial steps for its activation [129].

The inhibitory activity of capsaicin on TGF-β1 is related to liver fibrosis, where TGF-β1 stimulates the activation of HSCs. However in this study, it was demonstrated that capsaicin inhibits the increases in TGF-β1 expression caused by the component used to induce hepatic fibrosis in the rats used for this experimentation and demonstrated that capsaicin can in fact inhibit and partially block the TGF-β1/Smad signaling pathway [130].

### 6.3. Other Beneficial Impacts of Capsaicin

The anti-obesity property of capsaicin has been associated with uncoupling protein (UCP) proteins, which induce activity on energy expenditure and increase thermogenesis, which initially begins with the interaction of capsaicin with TRPV1, initiating the transmission of signals for UCP proteins [131]. However, there are other approaches where TRPV1 is not needed to have this anti-obesity activity. Mice with a TRPV1 knockout confirmed that capsaicin can lower lipid levels; this was caused by having a lower abundance of *Helicobacter*, *Desulfovibrio* and *Sutterrella,* Gram-negative bacteria that are responsible for lipopolysaccharides and also showed that TLR4 levels decreased, thus inflammation also lowered when there were low levels of lipopolysaccharides caused by the decreased abundance of those specific bacteria. Another different approach is the preconceptional capsaicin intervention, where the offspring of high-fat diet-induced obesity mice resulted in an improvement in metabolic disorders and the activation of brown adipose tissue, which establishes it as a potential strategy to prevent the inheritance of poor metabolic health [101,132].

Capsaicin has also been proven to alleviate redox imbalance or oxidative stress, thanks to its antioxidative activity. A mechanism that functions with capsaicin to reduce this oxidative stress in the intestines is the activation of the TRPV1/Protein Kinase A (PKA)/UCP2 pathway by elevating their gene expression [133]. Another similar study looked at alleviating redox imbalances. However, this was via circadian clock mechanisms. Their results were that capsaicin regulated a gene related to the circadian clock, *Bmal1* and they also managed to inhibit reactive oxygen species production and mitochondrial dysfunction in HepG2 cells [134].

Lastly, capsaicin has been observed as well in the effects on some mental diseases or disorders, such as Alsheimer’s disease, attenuating neurodegeneration in mice by reducing amyloid-beta levels via the promotion of non-amyloidogenic processing of amyloid precursor protein [135]. Also, the possible benefits of capsaicin on attention deficit hyperactivity disorder (ADHD) was evaluated, seeing that it can alleviate their symptoms in relation to gut microbiota, seeing that the change in abundance of certain bacteria could be beneficial in this manner; however, it still requires more investigation in depth with a larger group of individuals from all ages [111]. In Table 1, we list a series of effects from the treatment of capsaicin and derivatives.

### 6.4. Negative Impacts Caused by Capsaicin Consumption

As previously mentioned, capsaicin has been referred to as a “double-edged sword”, not only because it has been mentioned that high capsaicin consumption can have negative health effects such as gastric ulcers, gut inflammation, hyperalgesia, and the promotion of tumor development [117]. This last aspect makes it of critical attention. As mentioned earlier in this section, capsaicin acts as an anticancer nutraceutical with antiproliferative activities, but some of the literature points out that capsaicin may not be as good as it has been associated, especially when it is being consumed at a high dose. For example, supplying a high-capsaicin diet to mice, they achieved that a long-term intake of a high dose of capsaicin may contribute to the progression of gastric cancer and even hepatic metastases compared to the mice that were put on a normal dose of capsaicin. Additionally, it was demonstrated that the alteration of the microbiota caused by capsaicin increased the abundance of *Clostridiales* and *Firmicutes* (*Firmicutes*), which increased the peripheral 5-hydroxytryptamine levels, which aggravates metastasis [142]. However, according to the literature in which several studies around capsaicin consumption and risk of cancer were compared, there is not strong evidence that moderate consumption of capsaicin causes gastric cancer or any type of cancer [11,143]. Although there is evidence that capsaicin may be a risk factor, this can be caused by a misconception of the available information; this evidence is related to the dose that is being supplied.

## 7. Conclusions

Capsaicin represents a multifunctional phytochemical whose effects on host physiology are shaped by a complex interplay between its metabolic transformations, bioavailability, and interactions with the gut microbiota. Throughout this review, we have highlighted how capsaicin undergoes rapid hepatic metabolism and region-specific biotransformation in the brain, as well as how it may be enzymatically modified or reactivated by intestinal microbes. These processes not only determine its pharmacokinetic behavior but also influence its downstream biological activity. Capsaicin exhibits diverse systemic effects, including the modulation of neurotransmitter signaling, thermogenesis, redox balance, and gene expression patterns related to apoptosis, lipid metabolism, and epigenetic regulation. Importantly, capsaicin intake alters the composition and function of the gut microbiome, favoring short-chain fatty acid-producing and anti-inflammatory taxa while reducing potentially harmful microbial groups. These bidirectional host–microbiota interactions suggest that capsaicin may act as both a metabolic modulator and a selective microbiota-shaping agent. However, discrepancies across studies—including dose- and sex-dependent effects, as well as contradictory findings in cancer and inflammation models—highlight the importance of contextual factors such as baseline microbiota composition, genetic background, diet, and route of administration.

Future research integrating metabolomic, transcriptomic, and microbiome-resolved approaches is needed to elucidate the molecular mechanisms and ecological principles governing capsaicin’s effects. Clarifying these dynamics will be essential for advancing capsaicin as a dietary component or therapeutic agent in metabolic, neurological, and inflammatory diseases.

## Figures and Tables

**Table 1:** Beneficial and adverse effects of capsaicinoid treatment on different models.

Model	Effect	Description	Reference
C57BL/6 Mice	Anti-obesity effect	Capsaicin modulated the secretion of mucus in the gut from high-diet obesity-induced mice, which significantly contributed to the anti-obesity effects that capsaicin has, such as metabolism augmentation and energy expenditure.	[136]
C57BL/6J Mice	Anti-obesity effect	The effect of supplementation with capsaicinoids on mice fed with a high-fat-high-fructose diet reversed the obesity caused by this diet; additionally, it reduced hepatic lipid accumulation.	[137]
Primary Human Umbilical Vein Endothelial Cells	Anti-inflammatory activity	Capsaicin and hydrocapsaicin cardiovascular benefits were tested using primary human endothelial cells.	[138]
	Antioxidant activity		
MSTO-211H, NCI-H2052, NCI-H2452 and NCI-H28 Human Malignant Mesothelioma cell lines	Anticancer activity	Capsaicin demonstrated the ability to inhibit malignant mesothelioma (MM) cel0l growth by inducing S-phase cell cycle arrest, reducing motility and migration of MM cells, and inhibiting AKT and ERK1/2 activation.	[139]
MCF7 and MDA-MB-231 cell lines	Anticancer activity	Capsaicin-loaded nanoliposomes improved anticancer activities significantly on the different cell lines in comparison with capsaicin alone.	[140]
K562 cell line			
PANC1 cell line			
A375 cell line			
Human	Abdominal pain	Data from the Korean population were analyzed, showing that even low doses of capsaicin can cause abdominal pain. On the other hand, the risk of gastric cancer still requires evaluation, but high-level consumers had major risks and were susceptible to *H. pylori* infection.	[141]
	Gastric cancer risk		

## Data Availability

No new data were created or analyzed in this study.
